# B7-H3 combats apoptosis induced by chemotherapy by delivering signals to pancreatic cancer cells

**DOI:** 10.18632/oncotarget.20421

**Published:** 2017-08-24

**Authors:** Dongbao Li, Jun Wang, Jian Zhou, Shenghua Zhan, Yang Huang, Fei Wang, Zixiang Zhang, Dongming Zhu, Hua Zhao, Dechun Li, Gang Chen, Xinguo Zhu, Xin Zhao

**Affiliations:** ^1^ Department of General Surgery, The First Affiliated Hospital of Soochow University, Suzhou, China; ^2^ Pancreatic Disease Research Center, The First Affiliated Hospital of Soochow University, Suzhou, China; ^3^ Department of HBP, Suzhou Dushuhu Public Hospital, Soochow University Multi-Disciplinary Polyclinic, Suzhou, China; ^4^ Jiangsu Key Laboratory of Clinical Immunology, Soochow University, Suzhou, China; ^5^ Jiangsu Key Laboratory of Gastrointestinal Tumor Immunology, The First Affiliated Hospital of Soochow University, Suzhou, China; ^6^ Department of Emergency, The First Affiliated Hospital of Soochow University, Suzhou, China; ^7^ Department of Pathology, The First Affiliated Hospital of Soochow University, Suzhou, China; ^8^ Department of Hepatobiliary Surgery, The First Affiliated Hospital, Wenzhou Medical University, Wenzhou, China

**Keywords:** chemotherapy resistance, gemcitabine, Panc-1, pancreatic cancer, patu8988

## Abstract

**Objective:**

This study aimed to investigate the role of B7-H3 in chemotherapy resistance of pancreatic cancer cells and discover the potential signal transduction pathway and molecular targets involved.

**Methods:**

Immunohistochemical staining and real-time polymerase chain reaction (PCR) were used to determine the expression of B7-H3 in clinical specimens. Clinical data were applied to survival analysis. Phosphoprotein was purified from cultured Patu8988 cells using the Phosphoprotein Purification Kit. Cell apoptosis was detected using propidium iodide–Annexin V staining to investigate the relation between the expression of B7-H3 and Patu8988 cells treated with gemcitabine. Western blot was used to determine the effect of B7-H3 on the expression of proteins including extracellular signal–regulated kinase (ERK)1/2, epidermal growth factor receptor (EGFR), and Inhibitor of NF-κB(IκB) in Patu8988 cells; B7-H3 was activated by 4H7, which as an agonist monoclonal antibody to B7-H3.

**Results:**

The expression of B7-H3 was found to be higher in tumor tissues than in normal tissues of pancreatic carcinoma. Survival analysis revealed that patients in the low-B7-H3 expression group were likely to have a longer overall survival compared with those in the high-expression group (*P* < 0.05). B7-H3 activated by 4H7 could reduce gemcitabine-induced apoptosis in Patu8988 cells. Activation of B7-H3 by 4H7 induced variations in p-ERK1/2, EGFR, and IκB protein levels. When B7-H3 was upregulated, the expression levels of EGFR and p-ERK1/2 proteins significantly increased (*P* < 0.05), but the expression level of IκB significantly decreased (*P* < 0.05), especially in the gemcitabine-treated group.

**Conclusion:**

This study demonstrated that B7-H3 could deliver signals to pancreatic cancer cells to combat apoptosis induced by gemcitabine.

## INTRODUCTION

Recent advances in pancreatic surgery have the potential to improve outcomes for patients with pancreatic cancer [1]. However, the 5-year survival rate is only about 6%[2]. Moreover, this highly aggressive cancer is resistant to chemotherapy and radiation therapy [3]. Gemcitabine, the standard chemotherapy drug for advanced pancreatic cancer, has shown limited benefits because of profound chemoresistance [4]. As a first-line chemotherapeutic drug for pancreatic cancer, gemcitabine showed efficacy in less than 20% of treated patients [5]. Zhang *et al* [6] demonstrated a novel mechanism underlying acquired gemcitabine resistance in pancreatic cancer cells by inducing stemness via a Nox/ROS/NF-κB/STAT3 signaling pathway. Zhao *et al* [7] found that B7-H3 induced gemcitabine resistance in pancreatic carcinoma cells, at least partially, by downregulating survivin expression. However, the mechanism involved remains unclear. Therefore, it is urgent to elucidate the mechanisms by which chemoresistance occurs in patients with pancreatic cancer to achieve better therapeutic efficacy.

B7-H3, a novel member of the B7 family of costimulatory proteins, consists of two isoforms 2IgB7H3 and 4IgB7H3 [8]. The latter is more widely expressed in mature dendritic cells, T cells, and many human tumor cell lines including pancreatic cancer [9–11]. Zhang *et al* [12] declared that B7-H3 might promote U937 cell progression, and short hairpin RNA (shRNA) targeting B7-H3 significantly enhanced sensitivity to chemotherapeutic drugs. Zhang *et al* [13] showed that the overexpression of B7-H3 augmented anti-apoptosis of colorectal cancer cells by JAK2-STAT3. Many studies demonstrated that B7-H3 was closely correlated with chemotherapy resistance and apoptosis of pancreatic cancer cells. However, the mechanism of abnormal expression of B7-H3 in pancreatic cancer and its role in the changes in tumor biological behavior need to be further determined. Therefore, this study focused on the role of B7-H3 in chemotherapy resistance in pancreatic cancer cells to elucidate the signal transduction pathway and potential molecular targets involved.

## RESULTS

### Expression of B7-H3 was higher in tumor tissue than in a normal tissue of pancreatic carcinoma

**Figure 1:**
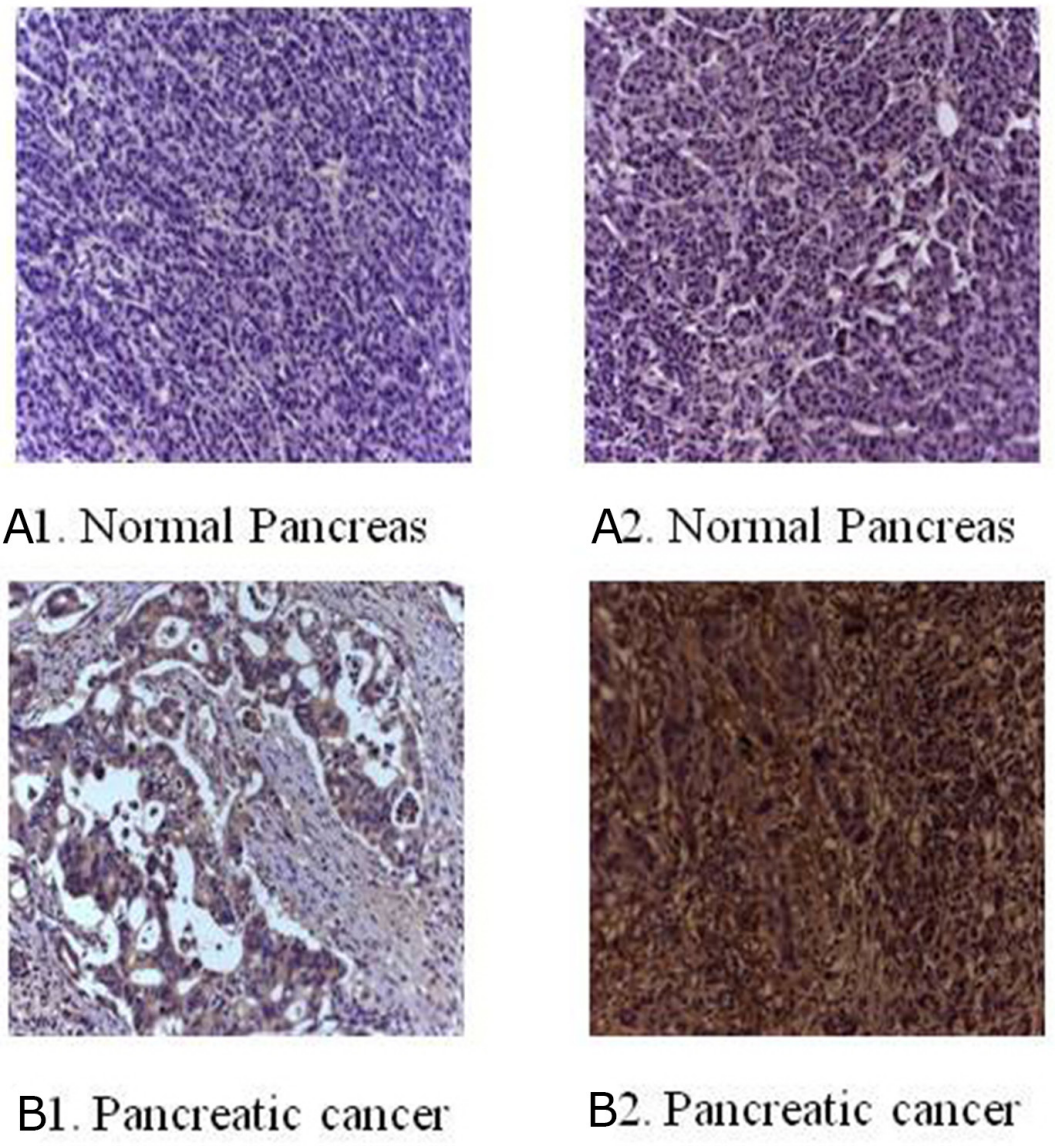
Immunohistochemical staining for B7-H3 in clinical specimens **(A1)** No expression in normal pancreas. **(A2)** Low expression in normal pancreas. **(B1)** Low expression in pancreatic cancer tissue. **(B2)** Overexpression in pancreatic cancer tissue (magnification, 200×).

**Figure 2:**
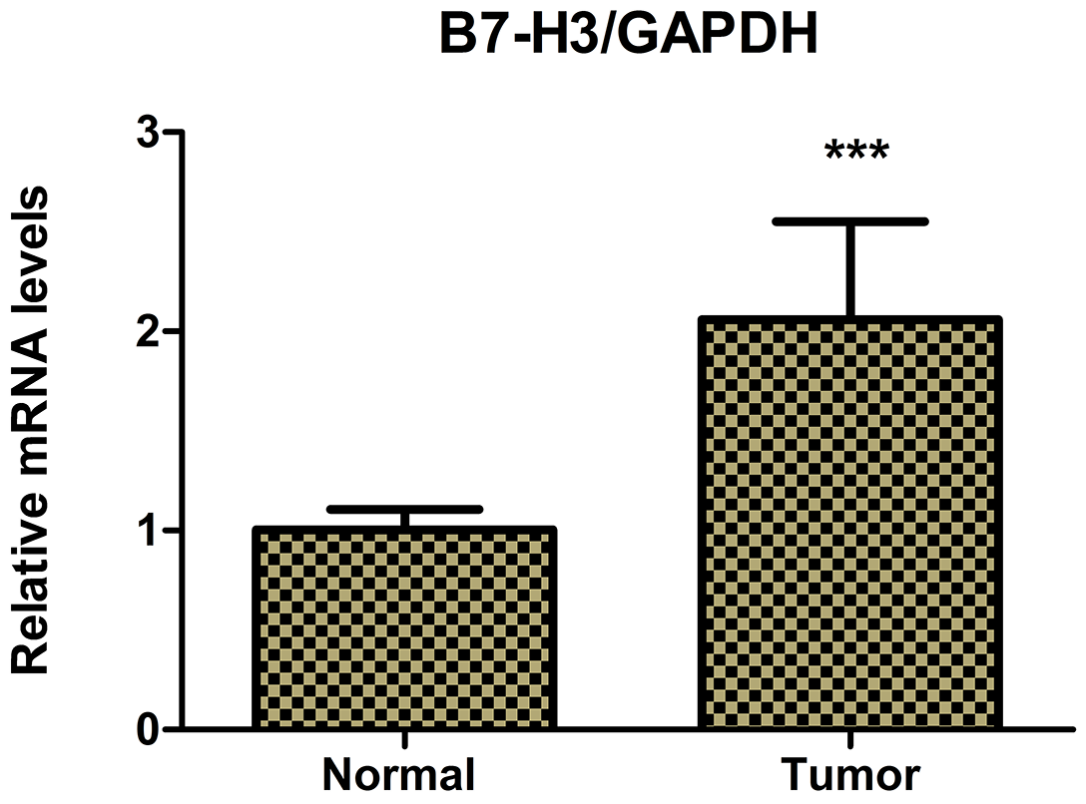
Real-time PCR determined the relative mRNA expression levels of B7-H3 in pancreatic carcinoma tissues and adjacent normal pancreatic tissues of 42 patients with pancreatic carcinoma The relative mRNA expression levels of B7-H3 were markedly higher in pancreatic carcinoma tissues than in adjacent normal pancreatic tissues (***P < 0.001).

The immunohistochemical staining method was used to detect the expression of B7-H3 in clinical surgical specimens of 42 patients with pancreatic carcinoma. The results revealed that B7-H3 was significantly overexpressed in the tumor tissue. Also, 19 tumor specimens showed high expression of B7-H3 (45.24%), 6 showed low expression of B7-H3 (14.29%), and 17 showed no expression (40.48%) (Figure 1). Moreover, real-time PCR determined the relative mRNA expression levels of B7-H3 in pancreatic carcinoma tissues and adjacent normal pancreatic tissues of the patients. The result showed that the relative mRNA expression levels of B7-H3 were markedly higher in pancreatic carcinoma tissues than in adjacent normal pancreatic tissues (*P* < 0.001) (Figure 2).

### Survival analysis

**Figure 3:**
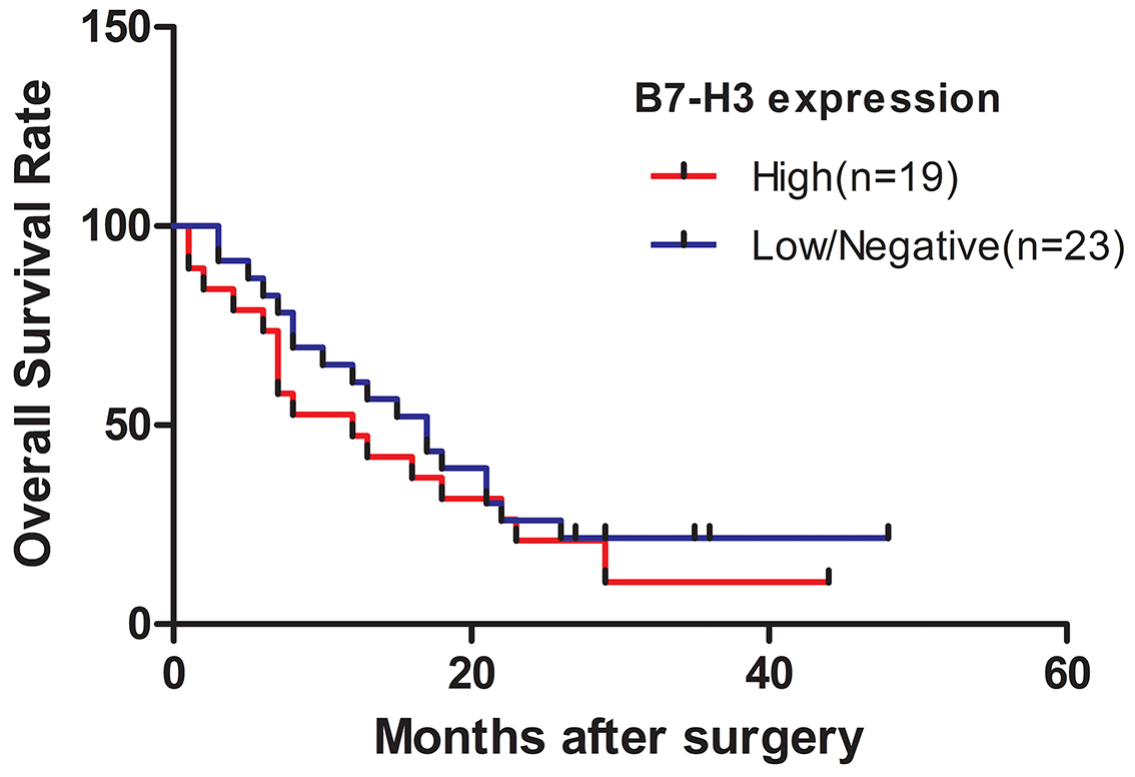
Survival curve of patients with pancreatic cancer stratified according to the expression levels of B7-H3 Patients in the low-expression group were likely to have a longer overall survival compared with those in the high-expression group (*P* < 0.05).

The survival curve is presented in Figure 3. Thirty-five deaths occurred. The cumulative median survival in patients with high and low expression of B7-H3 after the surgery was 10 and 18 months, respectively. Patients in the low-expression group were likely to have a longer overall survival compared with those in the high-expression group (*P* < 0.05).

### Identification of phosphorylated B7-H3 protein in Patu8988 cells stimulated with 4H7

**Figure 4:**
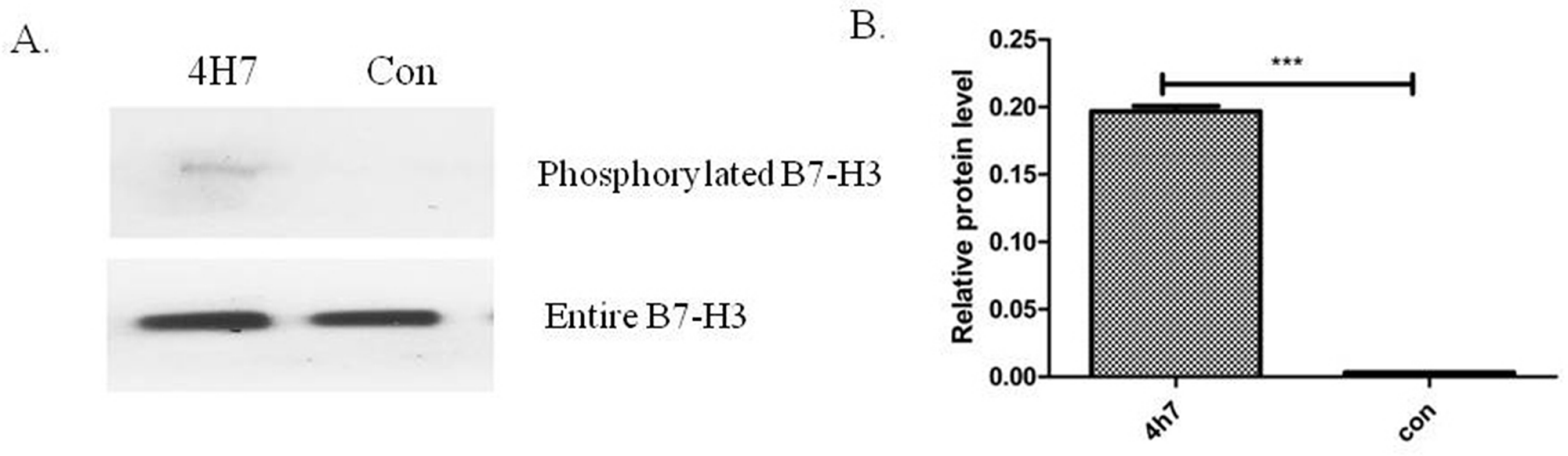
Phosphorylated B7-H3 protein was detected in the phosphoprotein fraction purified using a Qiagen Phosphoprotein Purification Kit in the Patu8988 cell line **(A)** The cells were divided into 4H7-stimulated group and control group. First, phosphorylated proteins were extracted from each group. Then, phosphorylated B7-H3 and entire B7-H3 were detected by Western blot. The results revealed that B7-H3 was phosphorylated after stimulation by 4H7. **(B)** The relative protein expression levels of phosphorylated B7-H3 were markedly high after stimulation by 4H7 (***P < 0.001).

The Qiagen Phosphoprotein Purification Kit was used to isolate phosphorylated proteins from the Patu8988 cell lysate. The cell lysate (Lfraction) was loaded on a column containing aphosphoprotein-binding resin, and the phosphorylated proteins were subsequently eluted (E fraction). Then, phosphorylated B7-H3 and entire B7-H3 were detected by Western blot with the anti-human B7-H3 antibody. The results revealed that B7-H3 was phosphorylated after stimulation with 4H7 (***P < 0.001) (Figure 4A and 4B), indicating signal conduction in the cells.

### Activation of B7-H3 reduced gemcitabine-induced apoptosis in Patu8988 cells

**Figure 5:**
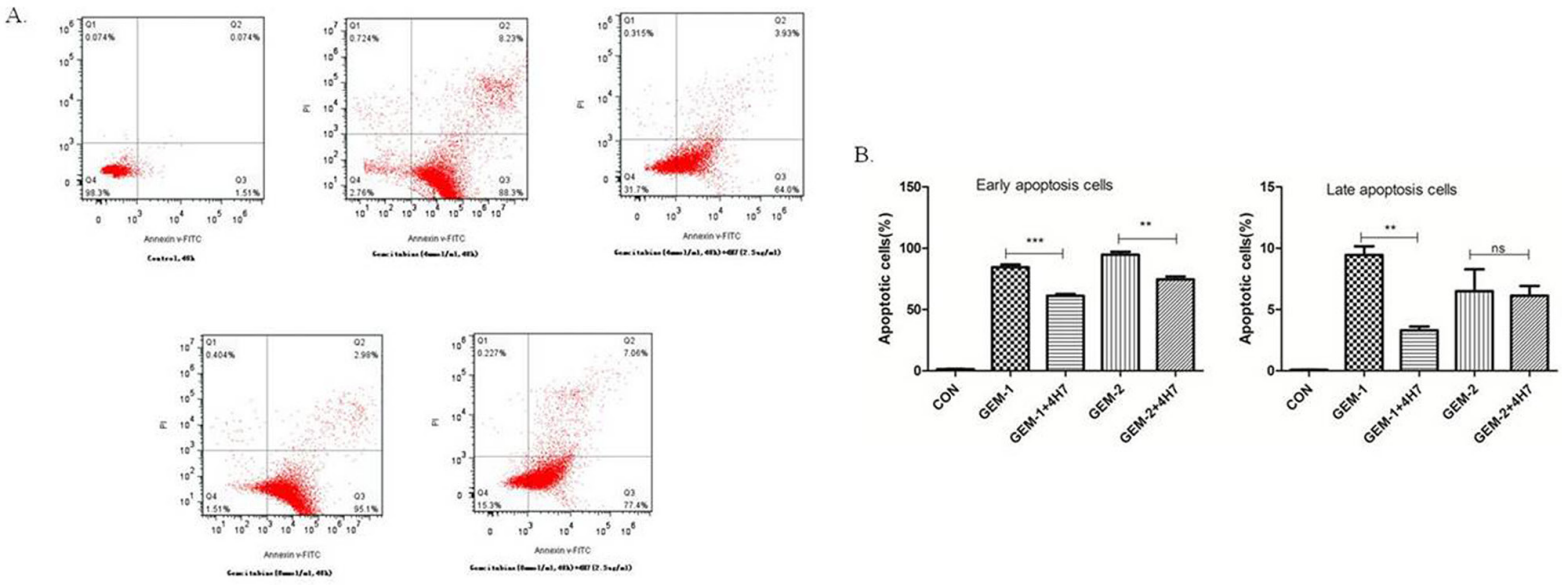
Flow cytometric analysis of Annexin V–FITC/PI to quantify gemcitabine-induced apoptosis in Patu8988 cells **(A)** The dot plots of Patu8988 cells treated with 4 and 8 mmol/mL of gemcitabine or 2.5 μg/mL 4H7 for 48 h are shown. **(B)** The early apoptosis increased when the dose of gemcitabine was improved, whereas it decreased after B7-H3 was activated by 4H7 (P < 0.05). This effect was not observed in late apoptosis. The results shown are representative of three independent experiments.

Gemcitabine exerts its cytotoxic effect by inducing tumor cell apoptosis. B7-H3 antibody 4H7 was developed in a previous study. Therefore, this study investigated the correlation between gemcitabine cytotoxicity observed in Patu8988 cells, in which B7-H3 was activated by 4H7 for 48 h, and its effects on apoptosis. After different treatments for 48 h, the early and late apoptosis of Patu8988 cells were measured separately using PI–Annexin V staining. The dot plots of Patu8988 cell apoptosis shown in Figure 5 exhibit that early apoptosis increased when the dose of gemcitabine was improved, whereas it decreased after B7-H3 was activated by 4H7 (*P* < 0.05). Hence, it could be concluded that activation of B7-H3 reduced gemcitabine-induced early apoptosis in Patu8988 cells.

### Activation of B7-H3 by 4H7 induced variations in p-ERK1/2, EGFR, and IκB protein levels

**Figure 6:**
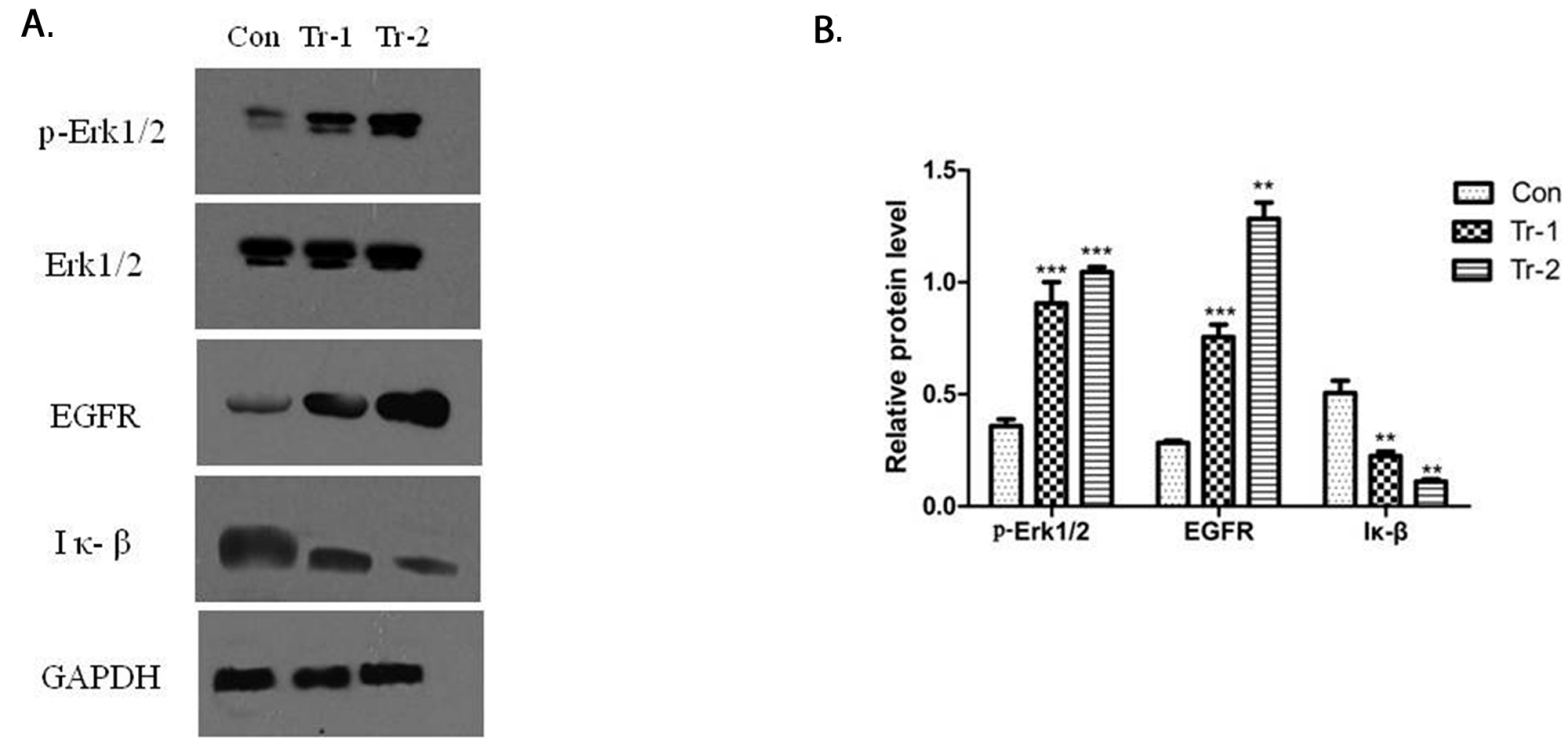
WT/Patu8988 cells were treated with different concentrations of 4H7 for 24 h **(A)** Western Blot was used to determine p-Erk1/2, Erk1/2, EGFR, and IκB protein levels. **(B)** The relative protein expression levels as determined by densitometric analysis of the Western blot results. Results are presented as mean ± standard deviation. The expression of p-Erk1/2 and EGFR was upregulated in a dose-dependent manner, whereas the expression of IκB was downregulated (con, control; Tr-1, 1 ng/mL 4H7; Tr-2, 5 ng/mL 4H7) (***P < 0.001; **P < 0.01).

The activation of B7-H3 can reduce gemcitabine-induced early apoptosis in Patu8988 cells. Hence, Western blot was used to determine the effect of B7-H3 on the expression of apoptosis proteins in Patu8988 cells. The cells were treated with 4H7 (1 and 5 ng/mL) for 24 h. The results showed that treatment with 4H7 led to variations in p-ERK1/2, EGFR, and IκB protein levels (Figure 6A). The expression of p-ERK1/2 and EGFR was upregulated in a dose-dependent manner, whereas the expression of IκB was downregulated (Figure 6B). This indicated that activation of B7-H3 by 4H7 induced variations in the levels of downstream molecules of ERK1/2, EGFR, and IκB, which might be associated with pancreatic cancer resistance to gemcitabine chemotherapy.

### Transfection efficiency of the lentivirus gene transfer vectors

**Figure 7:**
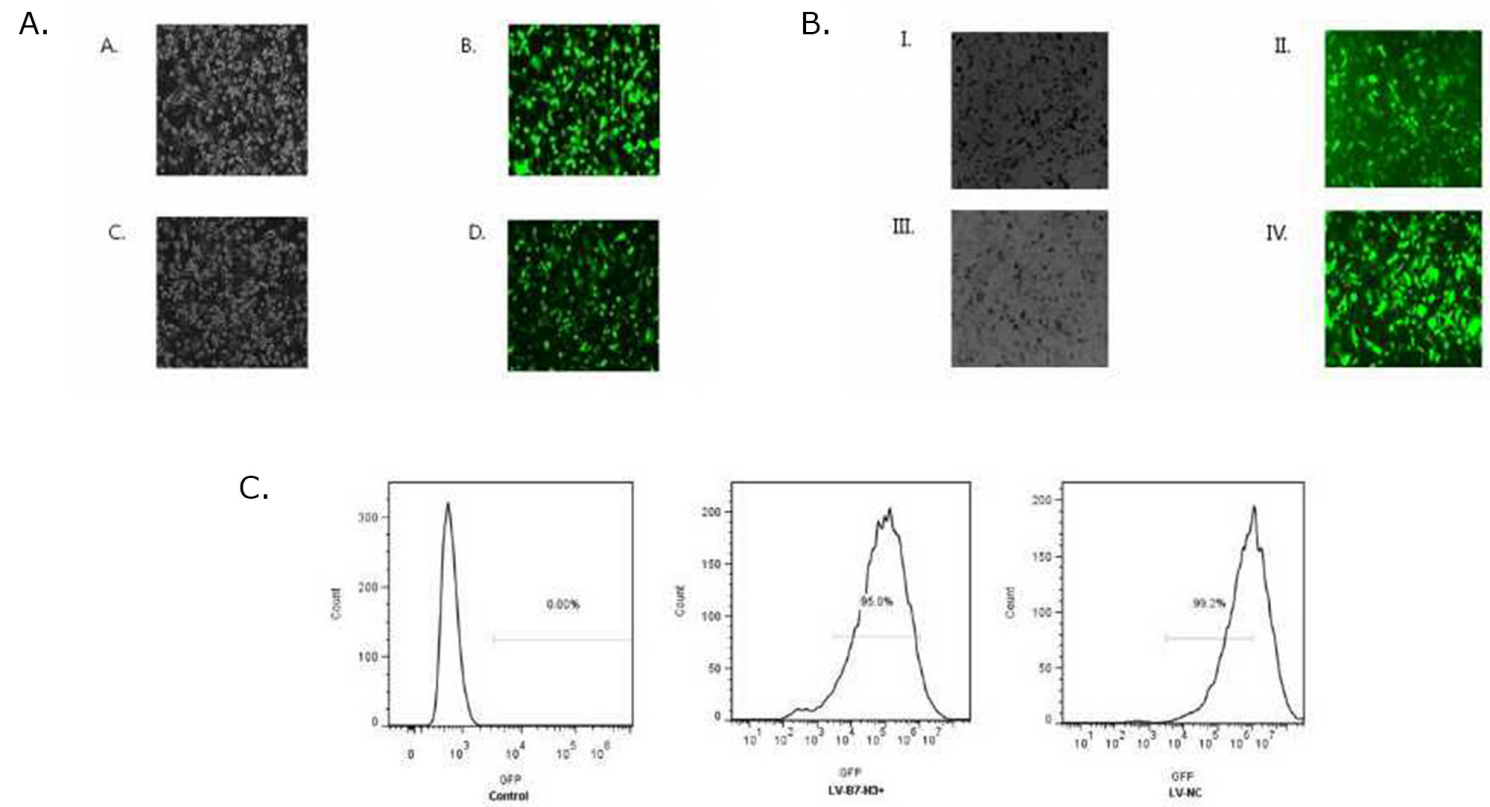
**(A)** Patu8988 cells displaying bright-field and green fluorescence (original magnification: 100×). (A and B) White light and green fluorescence of shB7-H3/Patu8988 cells. (C and D) White light and green fluorescence of LV-NC^-^/Patu8988 cells. **(B)** Panc-1 cells displaying bright-field and green fluorescence (original magnification: 100×). (I and II) White light and green fluorescence of LV-B7H3^+^/Panc-1 cells. (III and IV) White light and green fluorescence of LV-NC^+^/Panc-1 cells. **(C)** Expression of GFP was assessed by FCM. Infected positive cell ratio of LV-B7-H3^+^/Panc-1 and LV-NC^+^/Panc-1 cells was 95.0% and 99.2%, respectively.

Patu8988 and Panc-1 cells were observed under a fluorescence microscope 72 h after transfection with lentivirus gene transfer vectors (Figure 7A and 7B) and examined by FCM (Figure 7C). Infected positive cell ratio of LV-B7-H3^+^/Panc-1 and LV-NC^+/^Panc-1 cells was 95.0% and 99.2%, respectively. The result showed high efficiency of transfection.

### Expression of EGFR was positively associated with the expression of B7-H3 after cells were treated with gemcitabine

**Figure 8:**
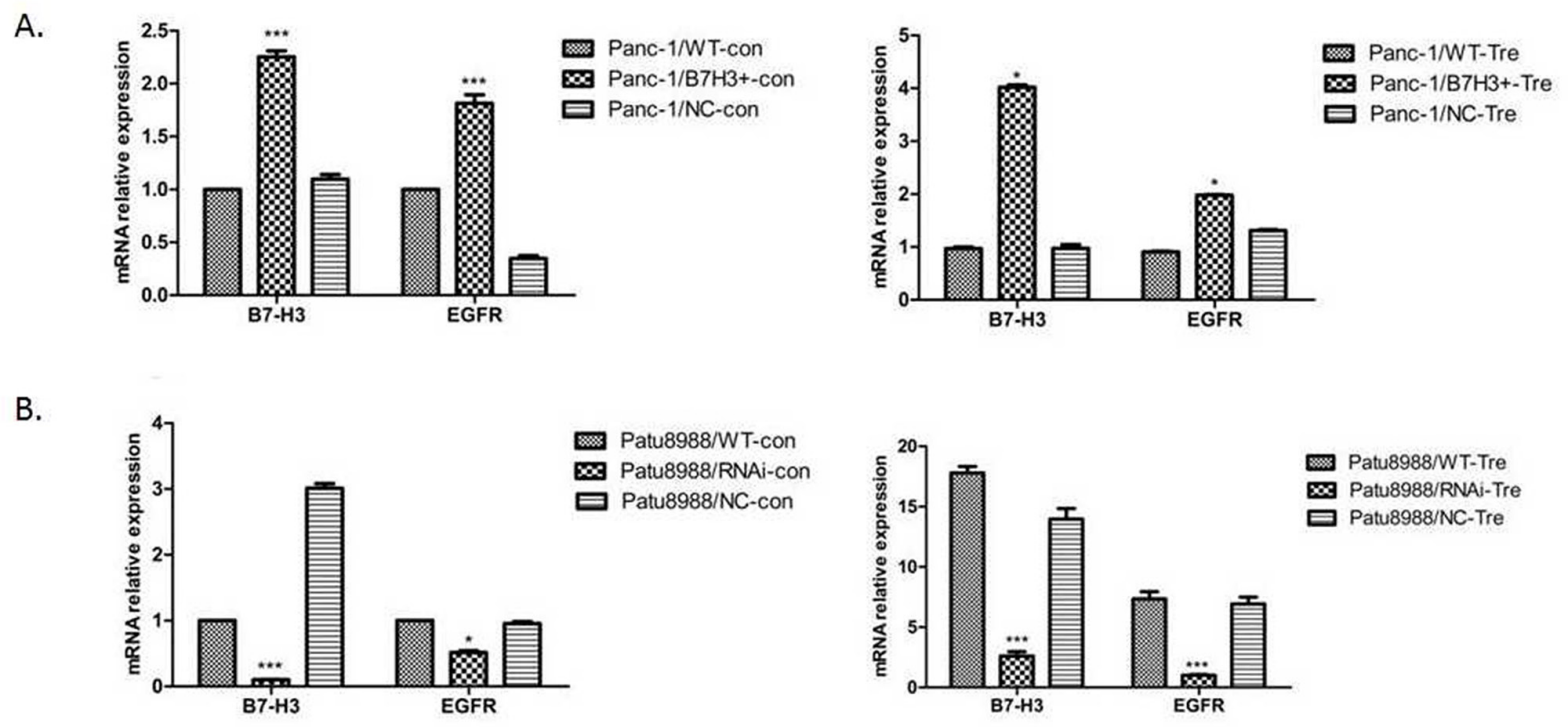
Relative mRNA expression of B7-H3 and EGFR was analyzed using the 2^-ΔΔCt^ method for different cell lines **(A)** Expression of B7-H3 increased in Panc-1/B7-H3+ cells compared with Panc-1/NC cells (P < 0.05), and the relative mRNA expression of EGFR also increased (P < 0.05). **(B)** Expression of B7-H3 decreased in Patu8988/B7-H3- cells compared with Patu8988/NC cells (P < 0.05), and the relative mRNA expression of EGFR also decreased (P < 0.05).

RT-PCR was performed to demonstrate the relative mRNA expression of B7-H3 and EGFR in Panc-1/NC, Panc-1/B7-H3^+^, Patu8988/NC, and Patu8988/B7-H3^-^ cell lines to investigate the relationship between B7-H3 and apoptosis in PC cell lines. The aforementioned cell lines were treated with gemcitabine (4 mmol/mL, 48 h). Both B7-H3 overexpression in Panc-1 cells and downregulation in Patu8988 cells affected the relative mRNA expression of EGFR (Figure 8A and 8B). The expression of B7-H3 increased in Panc-1/B7-H3^+^ cells compared with Panc-1/NC cells (*P* < 0.05), and the relative mRNA expression of EGFR also increased (*P* < 0.05). The expression of B7-H3 decreased in Patu8988/B7-H3^-^ cells compared with Patu8988/NC cells (*P* < 0.05), and the relative mRNA expression of EGFR also decreased (*P* < 0.05). Based on the data in this figure, that B7-H3 expression levels are positively associated with EGFR levels.

### Upregulation of B7-H3 slacked gemcitabine-induced apoptosis in Panc-1 cells

**Figure 9:**
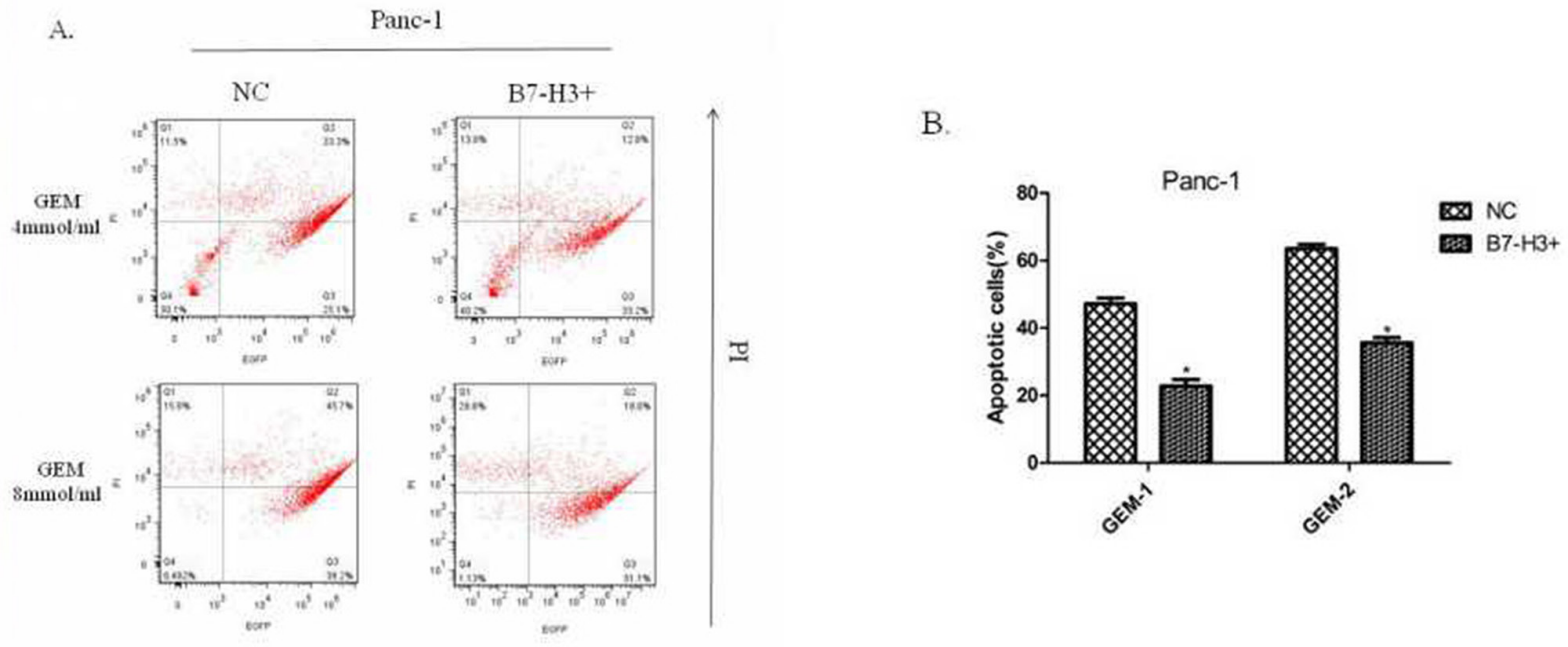
Upregulation of B7-H3 slacked gemcitabine-induced apoptosis in Panc-1 cells **(A)** Flow cytometry diagram at 72 h detected by FITC Annexin V/PI assay. **(B)** The percentage of PI-stained cells in Panc-1/NC and Panc-1/B7-H3+ cells. The results showed that the percentage of PI-stained cells in Panc-1/B7-H3+cells was less than the percentage in Panc-1/NC cells (Figure 8A and 8B) (P < 0.05).

A previous study showed that silencing of the expression of B7-H3 by shB7-H3 made the Patu8988 cells more prone to gemcitabine-induced apoptosis [7]. This study found the overexpression of B7-H3 in Panc-1/B7-H3^+^ cells and the control cell line Panc-1/NC, on treatment with gemcitabine (4 mmol/mL, 8 mmol/mL; 48 h, 72 h, respectively). Then, the PI–Annexin V Staining Apoptosis Detection Kit I manual was implemented to measure the percentage of PI-stained cells. The results showed that the percentage of PI-stained cells in Panc-1/B7-H3^+^cells was less than the percentage in Panc-1/NC cells (Figure 9A and 9B) (*P* < 0.05). Also, the response to gemcitabine was dose- and time-dependent. The figure only shows the result at 72 h. The results demonstrated that the upregulation of B7-H3 slacked gemcitabine-induced apoptosis in Panc-1 cells similar to Patu8988 cells.

### B7-H3 accommodated variation in EGFR protein to mediate pancreatic cancer cell resistance to gemcitabine-induced apoptosis

**Figure 10:**
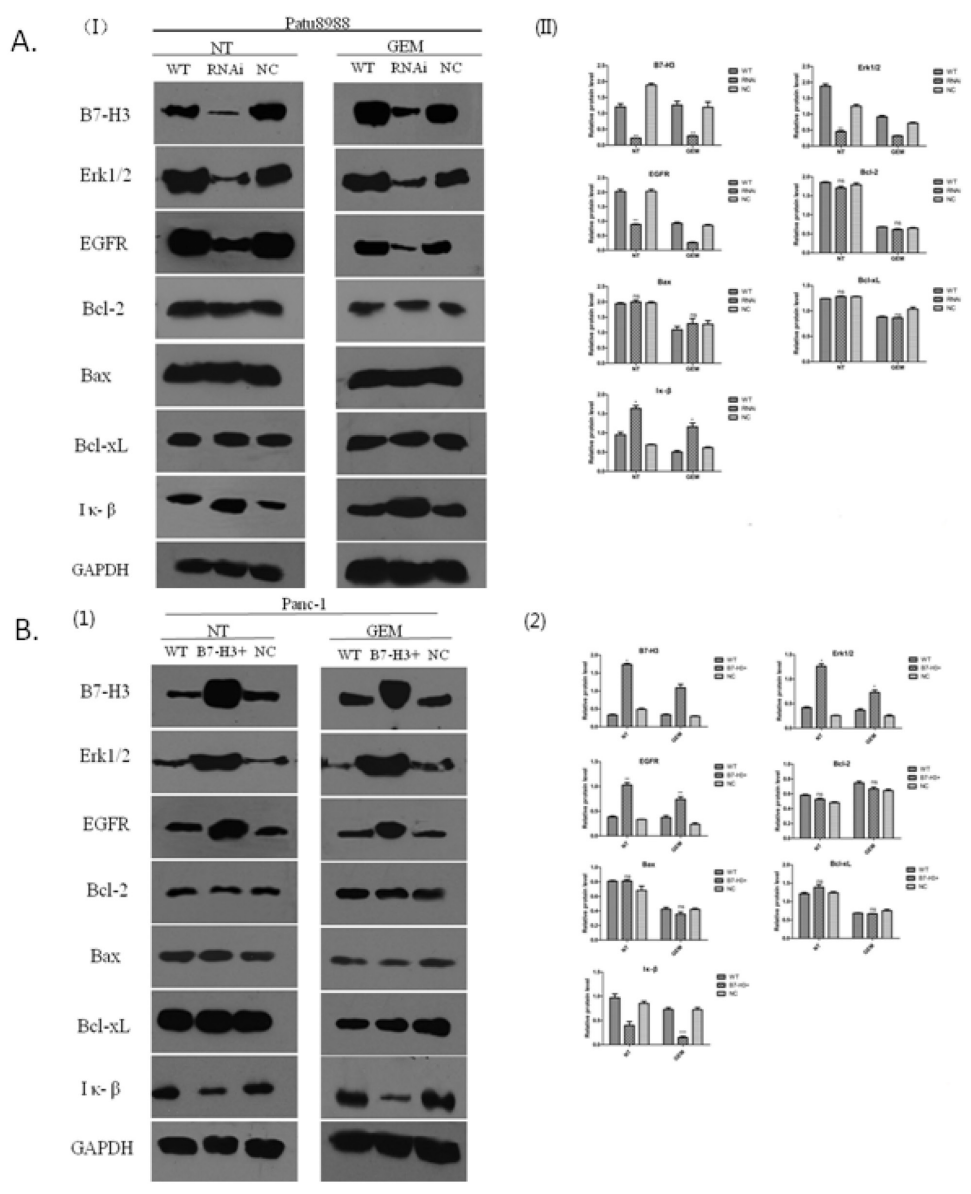
B7-H3 proteins of different cell lines demonstrated by Western blot analysis **(A)** Expression levels of Bcl-2, Bax, and Bcl-xL proteins had no obvious difference in Patu8988/WT, Patu8988/NC, and Patu8988/B7-H3- cell lines, regardless of gemcitabine treatment (P > 0.05). The expression levels of EGFR and ERK1/2 proteins significantly decreased (P < 0.05), and the expression level of IκB protein significantly increased (P < 0.05). **(B)** Expression levels of Bcl-2, Bax, and Bcl-xL proteins had no obvious difference in Panc-1/WT, Panc-1/NC, and Panc-1/B7-H3+ cell lines, regardless of gemcitabine treatment (P > 0.05). The expression levels of EGFR and ERK1/2 proteins significantly increased (P < 0.05), and the expression level of IκB protein significantly decreased (P < 0.05) (NT, No gemcitabine; GEM, gemcitabine, 8 mmol/mL, 48 h).

The expression of EGFR was associated with the expression of B7-H3 in the PC cells resistant to gemcitabine. The present study aimed to investigate the signaling pathway involved and potential molecular mechanisms. Western blot analysis was conducted to determine the relative protein level. Bcl-2, Bax, Bcl-xL, EGFR, ERK1/2, and IκB proteins were detected by Western blot and semi-quantitative analysis using the Image J software. Panc-1/WT, Panc-1/NC, Panc-1/B7-H3^+^, Patu8988/WT, Patu8988/NC, and Patu8988/B7-H3^-^ cell lines were treated with gemcitabine (8 mmol/mL, 48 h), while the control group received no treatment. Whole-cell lysates were used for detecting the expression levels of aforementioned proteins. The results revealed that the expression levels of Bcl-2, Bax, and Bcl-xL proteins had no obvious difference in Patu8988/WT, Patu8988/NC, and Patu8988/B7-H3^-^ cell lines, regardless of gemcitabine treatment (*P* > 0.05) (Figure 10A). However, when B7-H3 was downregulated, the expression levels of EGFR and ERK1/2 proteins significantly decreased (*P* < 0.05), and the expression level of IκB protein significantly increased (*P* < 0.05) (Figure 10A), especially in the gemcitabine-treated group after processing. Similar results were obtained in Panc-1cell lines. The results showed that the expression levels of Bcl-2, Bax, and Bcl-xL proteins had no obvious difference in Panc-1/WT, Panc-1/NC, and Panc-1/B7-H3^+^ cell lines, regardless of gemcitabine treatment (*P* > 0.05) (Figure 10B). However, when B7-H3 was upregulated, the expression levels of EGFR and ERK1/2 proteins significantly increased (*P* < 0.05), and the expression level of IκB protein significantly decreased (*P* < 0.05) (Figure 10B), especially in the gemcitabine-treated group. These results indicated that B7-H3 could accommodate variations in EGFR protein to mediate pancreatic cancer cell resistance to gemcitabine-induced apoptosis.

## DISCUSSION

This novel study confirmed that the expression of B7-H3 was higher in tumor tissues than in normal tissues of pancreatic carcinoma. Other studies also showed that the expression of B7-H3 was significantly associated with poor outcome in colon cancer [14]. Wu *et al* [15] reported that the expression of B7-H3 was related to survival time in gastric cancer cases. Zhao *et al* [16] discovered that the overexpression of B7-H3 in pancreatic cancer promoted tumor progression. Zhang *et al* [17] found that circulating B7-H3 was a valuable biomarker for nonsmall cell lung cancer (NSCLC), and an elevated level of circulating B7-H3 suggested a poor clinical outcome for NSCLC. These results suggested that B7-H3 had a critical role in tumor progression. Furthermore, 42 patients with pancreatic carcinoma were followed, and the survival analysis showed that the patients in the low-B7-H3 expression group was likely to have a longer overall survival compared with those in the high-B7-H3 expression group (*P* < 0.05).

B7-H3, identified in 2001, is a type I transmembrane protein that shares 20%–27% amino acid identity with other B7 family members [18]. Human B7-H3 possesses an isoform, the so-called 4Ig B7-H3 that contains a nearly exact tandem duplication of the IgV–IgC domain [19]. Previous studies, through the analysis of the amino acid sequence of B7-H3, found that the intracellular segment consisted of 45 amino acids (aa271–316) and higher-potential serine phosphorylation sites, besides two casein kinase II phosphorylation sites. B7-H3 antibody 4H7 was used to stimulate B7-H3, confirming that B7-H3 could be phosphorylated. Therefore, it was speculated that B7-H3 could influence the biological function of pancreatic cancer cells by transmitting signals in the cells.

The occurrence and development of tumors have a close relationship with apoptosis disorders [20]. Zhang *et al* [13] found that the overexpression of B7-H3 induced resistance to apoptosis in colorectal cancer cell lines by upregulating the JAK2–STAT3 signaling pathway. Zhao *et al* [7] demonstrated that silencing of B7-H3, through the lentivirus-mediated delivery of stable shRNA, was observed to increase the sensitivity of the human pancreatic carcinoma cell line Patu8988 to gemcitabine as a result of enhanced drug-induced apoptosis. The present study found that B7-H3 activated by 4H7 could reduce gemcitabine-induced apoptosis in Patu8988 cells. However, the mechanism is not clear.

Targeted therapies have been increasingly evaluated in patients with metastatic pancreatic cancer. Nearly 60% of pancreatic cancers demonstrate overexpression of EGFR [21], and therefore EGFR constitutes a promising therapeutic target. More recently, a multicenter trial evaluated the efficacy of cetuximab, an anti-EGFR monoclonal antibody, in combination with gemcitabine and failed to identify improved outcomes [22–24]. Lee *et al* [25] demonstrated that EGFR-mediated signaling in K-ras mutant pancreatic cancer cells phosphorylated ERK in a Ras-independent fashion, bypassing G proteins and Ras signaling. However, the correlation between B7-H3 and EGFR was not reported. The present study found that B7-H3 activated by 4H7 could induce variations in p-ERK1/2, EGFR, and IκB protein levels in Patu8988 cells. The expression of p-ERK1/2 and EGFR was upregulated in a dose-dependent manner, whereas the expression of IκB was downregulated. NF-κB is the most important transcription factor in inflammatory pathways that play major roles in tumorigenesis and thus can be considered targets for cancer prevention and therapy [26]. NF-κB, a key mediator of inflammatory response, plays a significant role in carcinogenesis and is now emerging as a link between inflammation and cancer. Inhibition of constitutive NF-κB, activity by a phosphorylation-defective IκBɑ (S32, 36A) (IκBɑM), suppressed pancreatic tumorigenesis in an orthotopic nude mouse model [27]. Tan *et al* [28] showed that metformin might inhibit pancreatic tumorigenesis by modulating multiple molecular targets in inflammatory pathways, including NF-κB/STAT3 inflammatory signaling. The present results indicated that B7-H3 might be associated with pancreatic cancer resistance to gemcitabine chemotherapy.

Based on the aforementioned results, pancreatic cancer cells with B7-H3 overexpression were knocked down in subsequent studies to identify possible proteins that mediated B7-H3 signaling resulting in chemotherapy resistance. It was found by RT-PCR that the expression of EGFR was associated with the expression of B7-H3 in the pancreatic cancer cell resistant to gemcitabine, and the upregulation of B7-H3 slacked gemcitabine-induced apoptosis in Panc-1 cells. The members of Bcl-2 protein family appear to be important factors in cell apoptosis [29]. Moreover, IκB was deemed to be related to cell apoptosis [30]. Therefore, Bcl-2, Bax, Bcl-xL, EGFR, Erk1/2, and IκB proteins were detected by Western blot and semi-quantitative analysis. When B7-H3 was upregulated, the expression levels of EGFR and ERK1/2 proteins significantly increased (*P* < 0.05), and the expression level of IκB significantly decreased (*P* < 0.05), especially in the gemcitabine-treated group. It has previously been shown that B7-H3 regulates the expression of Bcl-2, Bcl-xL, and Bax via the JAK2/STAT3 signaling pathway to increase the anti-apoptotic ability of cancer cells [13]. Also, Liu *et al* [31] showed that B7-H3 promoted cell migration and invasion through the JAK2/STAT3/MMP9 signaling pathway in colorectal cancer. However, the expression of Bcl-2, Bcl-xL, and Bax had no obvious difference, irrespective of B7-H3 upregulation or downregulation, in the present study. These results indicated that B7-H3 could accommodate the variations in EGFR to mediate pancreatic cancer cell resistance to gemcitabine-induced apoptosis.

In conclusion, the present study demonstrated that B7-H3 could deliver signals to pancreatic cancer cells to combat the apoptosis induced by gemcitabine. It also showed the molecular mechanisms underlying the aforementioned effect. These findings indicated a novel role of B7-H3 in the regulation of chemotherapy resistance of pancreatic cancer cells. B7-H3 may serve as a potential therapeutic target in the progression of pancreatic cancer.

## MATERIALS AND METHODS

### Reagents

B7-H3 (D9M2L) XP rabbit monoclonal antibody (mAb), Bcl-2(50E3) rabbit mAb, Bcl-xL (54H6) rabbit mAb, Bax (D2E11) rabbit mAb, EGF receptor (C74B9) (D9M2L) rabbit mAb, IκB-alpha (44D4) rabbit mAb, ERK rabbit mAb, p-ERK rabbit mAb and glyceraldehyde 3-phosphate dehydrogenase (GAPDH) rabbit mAb were purchased from Cell Signaling Technology (USA). 4H7(Jiangsu Key Laboratory of Clinical Immunology, Soochow University, Suzhou). The horseradish peroxidase–conjugated secondary anti-rabbit antibodies were purchased from Bio-Rad (CA, USA). Gemcitabine was purchased from Lilly France, Inc. (Neuilly, France). A Phosphoprotein Purification Kit was purchased from Qiagen Sample and Assay Technologies (Germany). Dulbecco’s modified Eagle’s Medium (DMEM) was purchased from Sigma–Aldrich (MO, USA). Fetal bovine serum (FBS) was supplied by Sijiqing Biological Manufacturer Co., Ltd (Hangzhou, China). The Annexin V–fluorescein isothiocyanate (FITC) Apoptosis Detection Kit was purchased from Miltenyi Biotec (Germany).

### Cells and cell culture

The pancreatic cancer cell lines Panc-1 and Patu8988 were purchased from the Chinese Academy of Science Cell Bank. Panc-1 cells were cultured in DMEM, and Patu8988 cells were cultured in RPMI-1640 medium. All media were supplemented with 10% FBS and 1% penicillin–streptomycin (Gibco-BRL) at 37°C in 5% CO_2_ atmosphere.

### Clinical specimens from patients and survival analysis

Clinical surgical specimens were obtained from 42 patients with pancreatic carcinoma who underwent surgery between October 2010and December 2012 at the First Affiliated Hospital of Soochow University. The diagnosis of pancreatic carcinoma was confirmed by histopathology. The patients did not receive preoperative adjuvant treatment before surgery. Tumor tissues were dissected, and paired adjacent normal pancreatic tissues were obtained from the same patients. The tissue specimens were immediately fixed in 10% buffered formalin for immunohistochemical estimation of expression of B7-H3 and then stored at -80°C until further use. Reverse transcription–polymerase chain reaction (RT-PCR) was performed to detect the expression of B7-H3 from 42 pancreatic carcinoma specimens. The clinical follow-up period of patients ranged from 1 to 60 months. Clinical data were applied to survival analysis. The overall survival time was defined as the interval from the date of diagnosis to pancreatic carcinoma–related death. This clinical study was approved by the ethics committee of The First Affiliated Hospital of Soochow University.

### Phosphoprotein purification from cultured Patu8988 cells

Patu8988 cells were preincubated with or without 4H7 for 30 min. Isolation of phosphoproteins from cellular extracts was carried out using the Phosphoprotein Purification Kit according to the manufacturer’s protocol. In short, a cell pellet corresponding to 10^7^ cells was resuspended in 5 mL of lysis buffer containing protease inhibitors and Benzonase Nuclease. The mixture was incubated for 30 min at 4°C and vortexed briefly every 10 min. The supernatant was harvested, and the protein concentration was determined. The protein concentration was measured in all eluted fractions to determine the most concentrated fraction. Then, Western blot was implemented with anti-human B7-H3 antibody to detect the phosphorylated B7-H3 protein.

### Evaluation of cell apoptosis by propidium iodide–annexin V staining

Cell apoptosis was evaluated by propidium iodide (PI)–AnnexinV staining to investigate the relation between the expression of B7-H3 and Patu8988 cells treated with gemcitabine. This study used B7-H3 antibody 4H7. Briefly, 1 × 10^5^/well Patu8988 cells were paved into six-well plates and cultured overnight. Then, the cells were assorted into five groups: control group, 4 mmol/mL gemcitabine group, 4 mmol/ mL gemcitabine + 2.5 μg/mL 4H7 group, 8 mmol/mL gemcitabine group, and 8 mmol/mL gemcitabine + 2.5 μg/mL4H7 group. The treatment lasted for 48 h. Subsequent to harvesting, the cells were resuspended in 100 μL of 1× binding buffer. Next, 10 μL of Annexin V–FITC was added, and the mixture was incubated in the dark for 15 min at room temperature. After washing and centrifuging cells, the cell pellet was resuspended in 500 μL of 1× binding buffer. Finally, 5 μL of the PI solution was added immediately prior to analysis by FACSCalibur flow cytometry (FCM) and FlowJo7.6 software. This experiment was repeated three times. After Panc-1 cells were transfected with B7-H3 lentivirus and NC lentivirus, the aforementioned method was used to detect the cell apoptosis status. B7-H3+/Panc-1 and NC/Panc-1 cells were separately treated with 4 and 8 mmol/mL gemcitabine for 48 and 72 h, respectively. Then, PI–Annexin V Staining Apoptosis Detection Kit I manual (Invitrogen Life Technologies, CA, USA) was implemented. However, it was that the green fluorescent protein (GFP) expression was capable of interfering with the FITC analysis assessed by flow cytometry, as both expressed a similar green fluorescence. Therefore, PI reagent was used only to detect apoptosis. This experiment was repeated three times.

### Lentivirus transfection of human pancreatic cancer cell lines Patu8988 and Panc-1

shRNA of the human B7-H3 (5'-CTCTGCCTTCTCACCTCTTTG-3') and overexpressing hB7-H3 of lentivirus gene transfer vectors encoding the GFP sequence were constructed by Shanghai Genechem Co. (Shanghai, China).

The control lentivirus gene transfer vectors for the downregulation and upregulation of hB7-H3, respectively, were the nontargeted control mock lentivirus (LV-NC^-^) and LV-NC^+^. Patu8988 cells were transfected with shRNA/B7-H3 and LV-NC^-^/B7-H3 lentivirus, and Panc-1 cells were transfected with LV-B7-H3^+^ and LV-NC^+^/B7-H3 lentivirus, according to the manufacturer’s instructions. After transfection, FCM, RT-PCR, and Western blot analysis were performed to confirm the downregulation and upregulation of B7-H3 mRNA and protein in Patu8988 and Panc-1 cell lines, respectively

### Quantitative real-time RT-PCR

The total RNA was isolated from cell lines using TRIzol, following the manufacturer’s instructions and quantified using a NanoDrop 2000 spectrophotometer (Thermo Scientific, MA, USA). The total RNA was treated with RNase-free DNase to remove residual genomic DNA. The first-strand cDNA was synthesized from 1.0 μg RNA using an oligo-dT primer and avian myeloblastosis virus reverse transcriptase. The real-time mRNA expression analysis of target genes (B7-H3 and EGFR) and a housekeeping gene (GAPDH) for control was performed using the SYBR Green Real-time PCR Master Mix and real-time PCR amplification equipment. The primer sequences were as follows: sense sequence (5' to 3') CTCTGCCTTCTCACCTCTTTG and antisense sequence (5' to 3') CCTTGAGGGAGGAACTTTATC for B7-H3; sense sequence (5' to 3') CCCACTCATGCTCTACAACCC and antisense sequence (5'to 3') TCGCACTTCTTACACTTGCGG for EGFR; and sense sequence (5'to 3') TGACTTCAACAGCGACACCCA and antisense sequence (5'to 3') CACCCTGTTGCTGTAGCCAAA for GAPDH. The PCR conditions consisted of one cycle at 95°C for 15 s, followed by 45 cycles at 95°C for 5 s and at 60°C for 30 s. The relative quantification was provided by the Ct values using the 2^-ΔΔCt^ method, determining the reactions for target genes and an internal control gene in all samples. Assays were triplicated, and the average expressions levels were determined.

### Immunohistochemistry staining analyses

Clinical specimens were used for immunohistochemical staining analyses. Paraffin-embedded tissues were cut into 4-μm sections and incubated with the rabbit anti-human B7-H3 polyclonal antibody (1:50 dilution) overnight at 4°C. SP-9000 Histostain-Plus kits (ZSGB-BIO, Beijing, China) were used according to the manufacturer’s instructions. The brown staining in the cytoplasm was read as positive reactivity for B7-H3. Scoring was measured according to the cell cytoplasm staining pattern: 0, no cytoplasmic staining;1, weak cytoplasmic staining; 2, moderate cytoplasmic staining; and 3, strong cytoplasmic staining.

### Flow cytometry

After the prescribed treatments, the cells were trypsinized, collected, and washed three times with phosphate-buffered saline (PBS). They were then fixed in 70% ice-cold ethanol for 2 h. They were resuspended in PBS at a concentration of 10^6^ cells/mL and then incubated with PI for 2 h at room temperature in the dark. Finally, the cells were analyzed by FACScan (Becton-Dickinson, CA, USA) using the FlowJo 7.6 software.

### Western blot analysis

The harvested cells were washed with PBS and lysed with cell lysis buffer on ice. The protein concentrations were tested using the Bicinchoninic Acid Protein Assay Kit. Equal amounts of protein samples were prepared and fractionated by electrophoresis using sodium dodecyl sulfate–polyvinylidene difluoride membrane. The membranes were blocked and incubated with primary antibodies at 4°C overnight. Then, they were washed three times with Tris-buffered saline and Tween 20 and incubated with a second antibody at room temperature for 2 h. Following further washing, the protein bands were subsequently detected by an enhanced chemiluminescence assay, with the exposure time according to the brightness of the band. The intensities of the bands were calculated by semi-quantitative analysis using the Image J software. Each experiment was repeated three times.

### Statistical analyses

Data are presented as mean (± standard deviation). Differences in mean values between groups were analyzed by one-way analysis of variance, and Student *t* test was used for comparing two groups. At least three independent experiments were performed for all the studies. Differences were considered to be statistically significant when *P* values were less than 0.05. The data were analyzed using GraphPad Prism 6 (GraphPad Software Inc., CA, USA).

## Abbreviations

B7-H3: B7 Homolog 3
DC: dendritic cell
FITC: fluorescein isothiocyanate
MOI: multiplicity of infection
EGFP: enhanced green fluorescent protein
FCM: flow cytometry
RT-PCR: real-time polymerase chain reaction
IFN-γ: Interferon-γ
EGFR: epidermal growth factor receptor
Bcl-2: B-cell lymphoma-2
